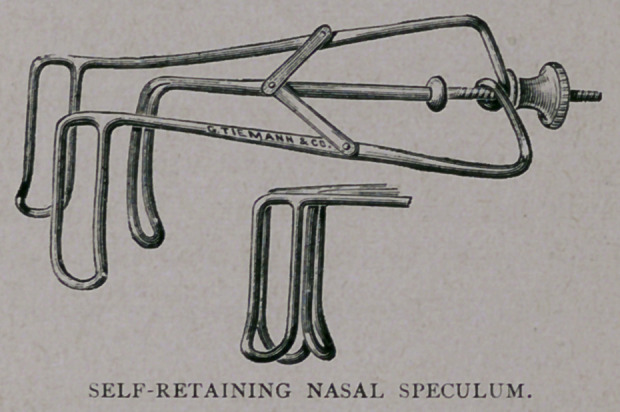# A Self-Retaining Nasal Speculum

**Published:** 1889-04

**Authors:** Frank Hamilton Potter

**Affiliations:** Buffalo, N. Y., Lecturer on Laryngology in the Medical Department of Niagara University; 273 Franklin Street


### New Instruments.

### A SELF-RETAINING NASAL SPECULUM.

By FRANK HAMILTON POTTER, M. D., Buffalo, N. Y.,

Lecturer on Laryngology in the Medical Department of Niagara University.

The accompanying cut conveys so clear an idea of this instrument, that very little description is necessary. It is so constructed as to be very light. It has three blades, of the Bosworth model. The blades can be opened and held at any point, by means of a nut and screw working on the middle bar, which, in turn, has attachments connecting

SELF-RETAINING NASAL SPECULUM.

with the outer bars. By this means, it can be adapted to any size of nostril without causing pain. It is self-retaining when properly dilated, and thus is of value in surgical procedures, allowing the operator the free use of both hands. It is made by Messrs. Tiemann & Co., of New York, and can be obtained of them.

273 Franklin Street.

## Figures and Tables

**Figure f1:**